# Supplementary figures and images for: WNK2 may promote ovarian cancer progression by upregulating POU5F1B
Source: PLoS One. 2026 Feb 2;21(2):e0332003. doi: 10.1371/journal.pone.0332003 (PMC12863568; doi:10.1371/journal.pone.0332003)

Fig2A:

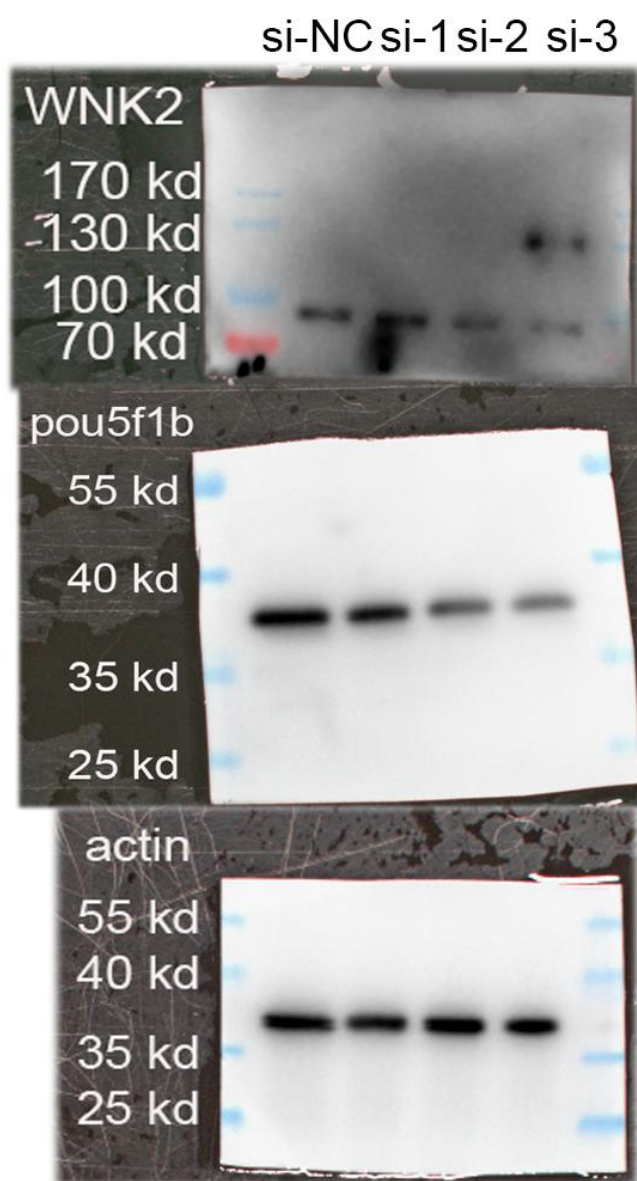

Fig2B:

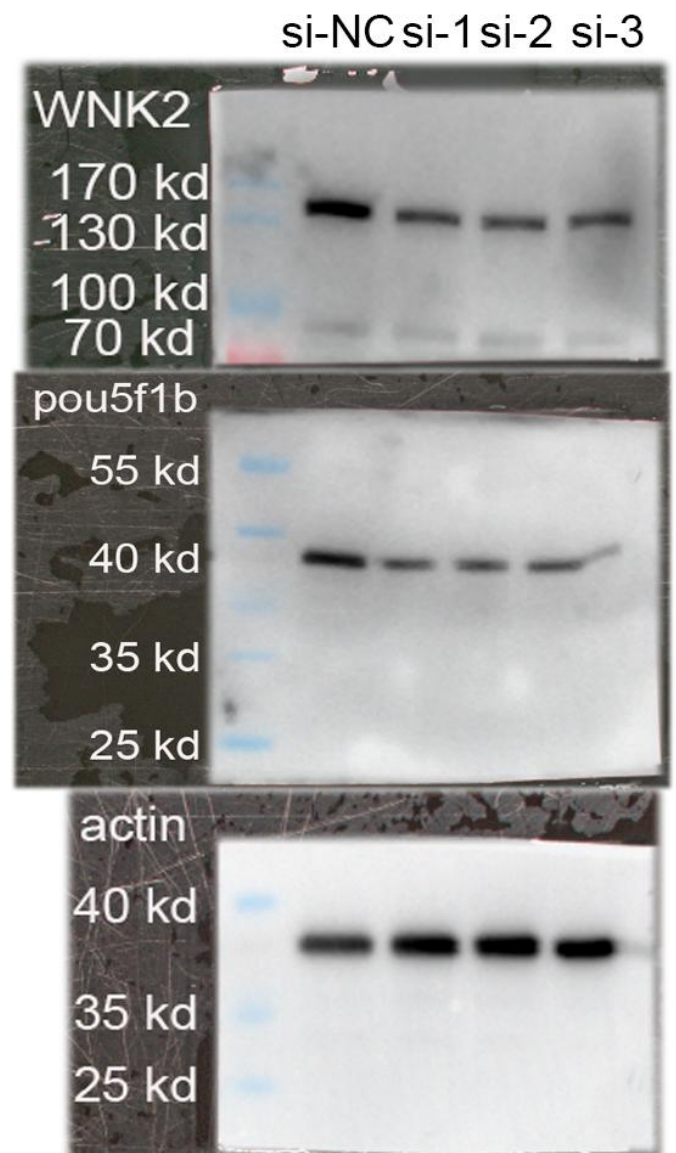

Fig2C:

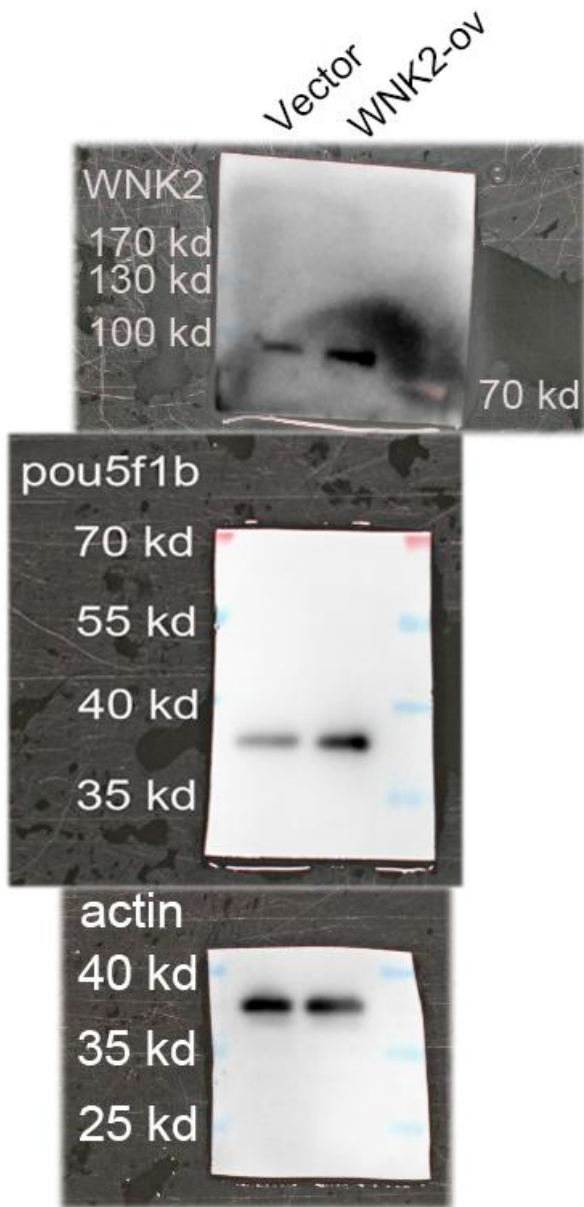

Fig2D:

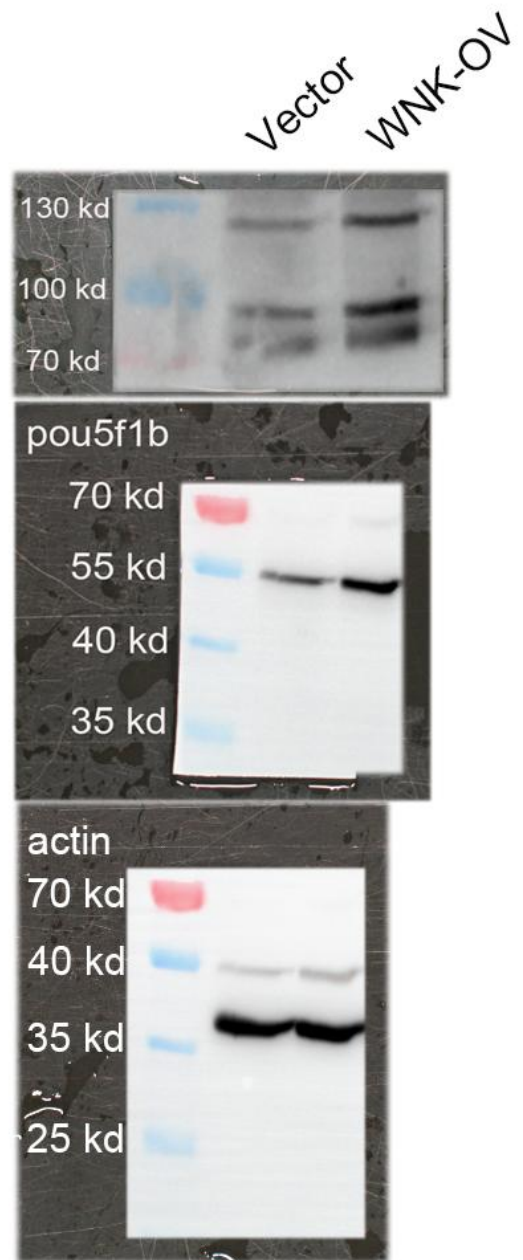

Fig4A:

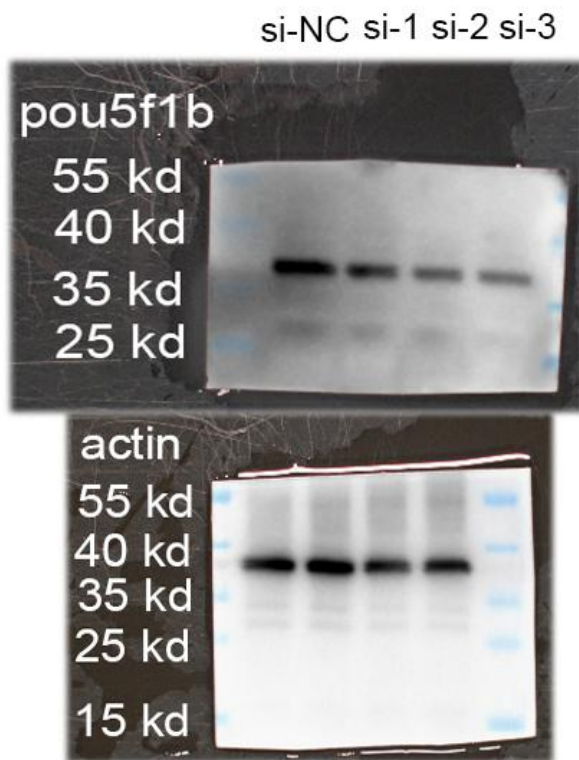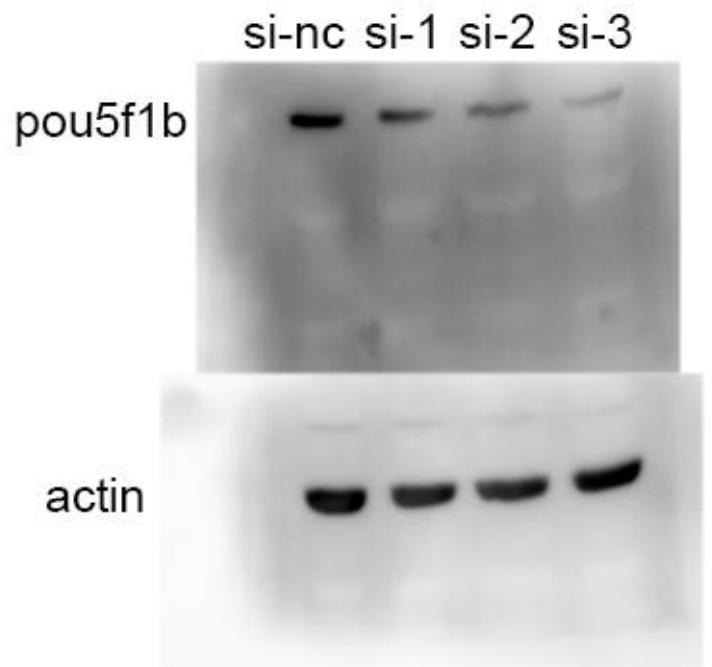

Fig5A:

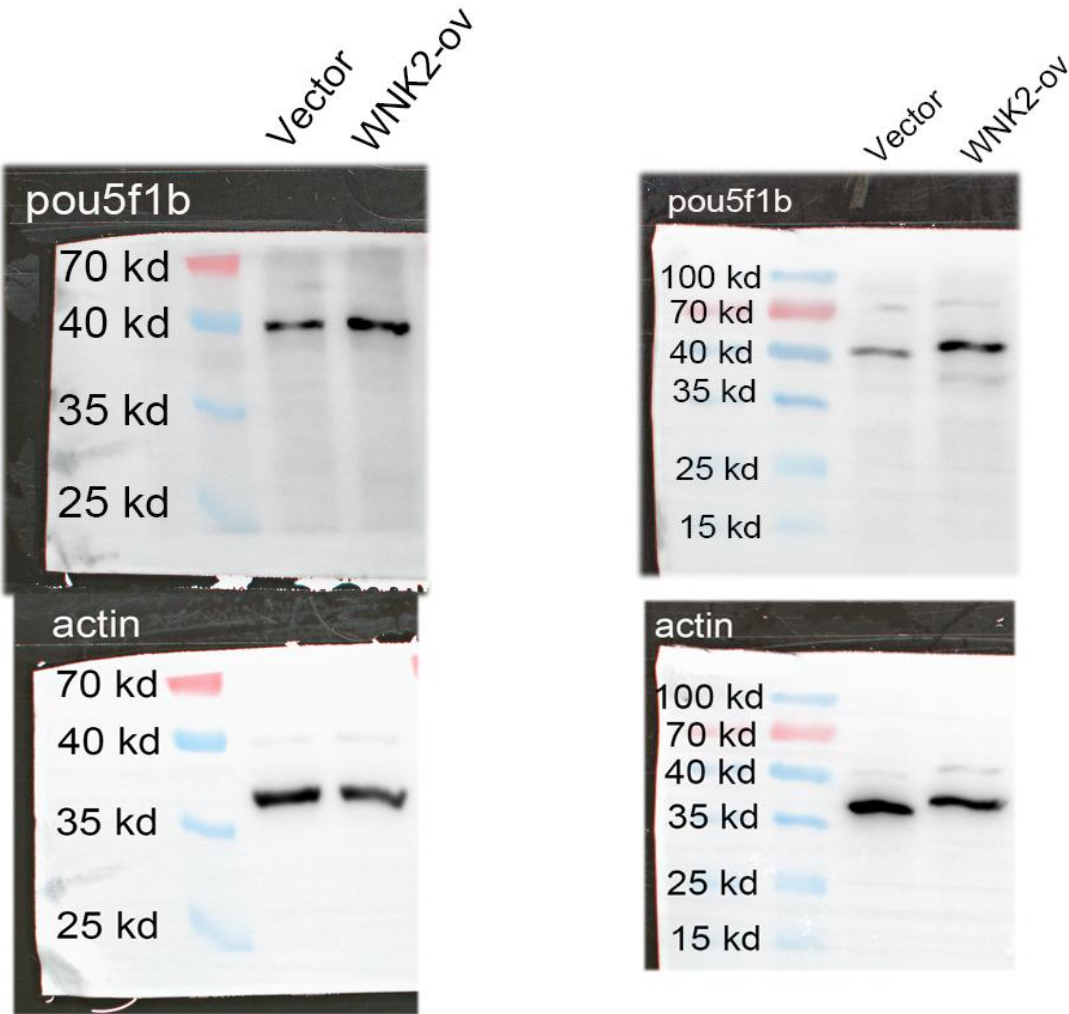

Fig6A-B:

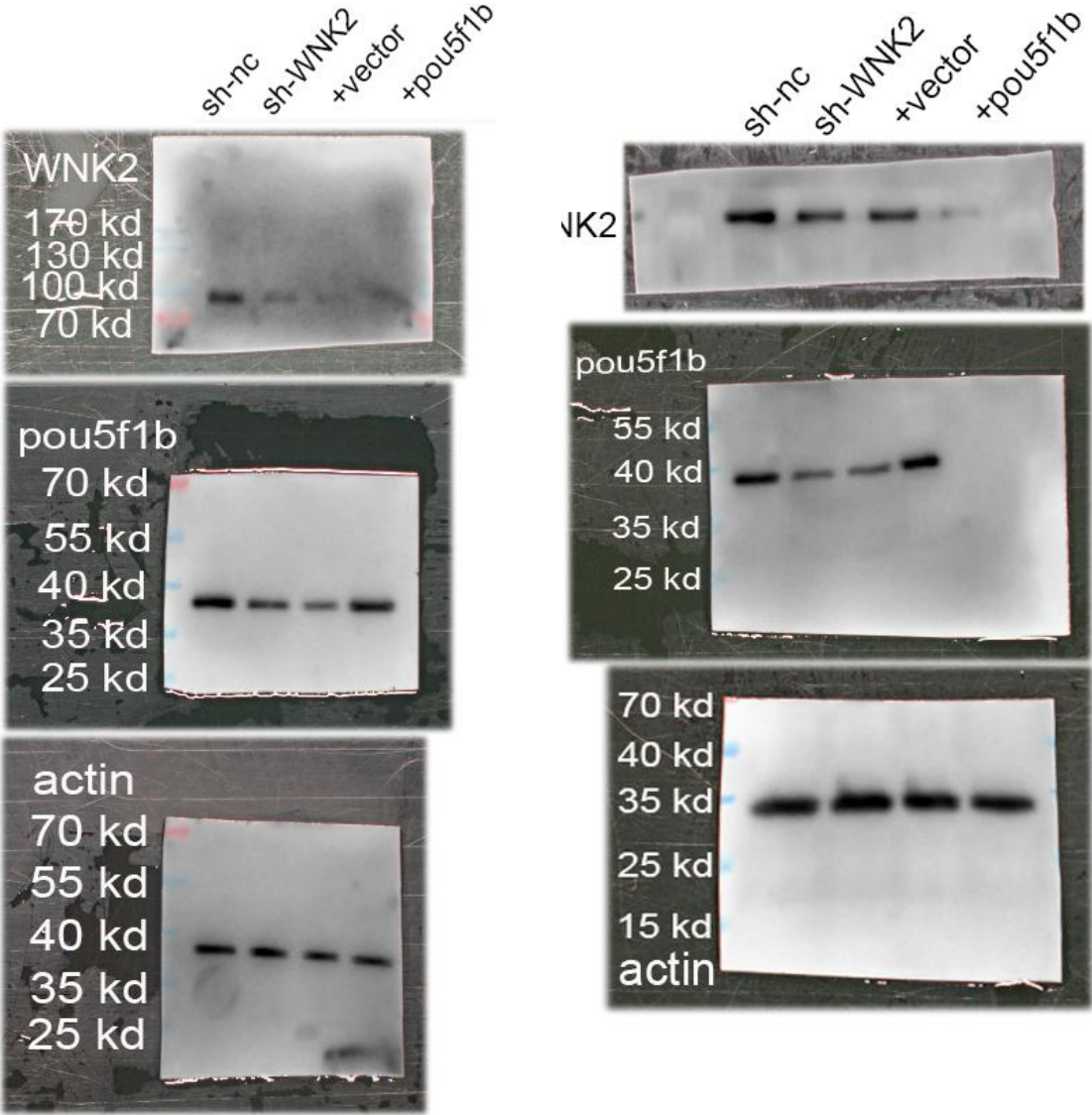

Fig6l:

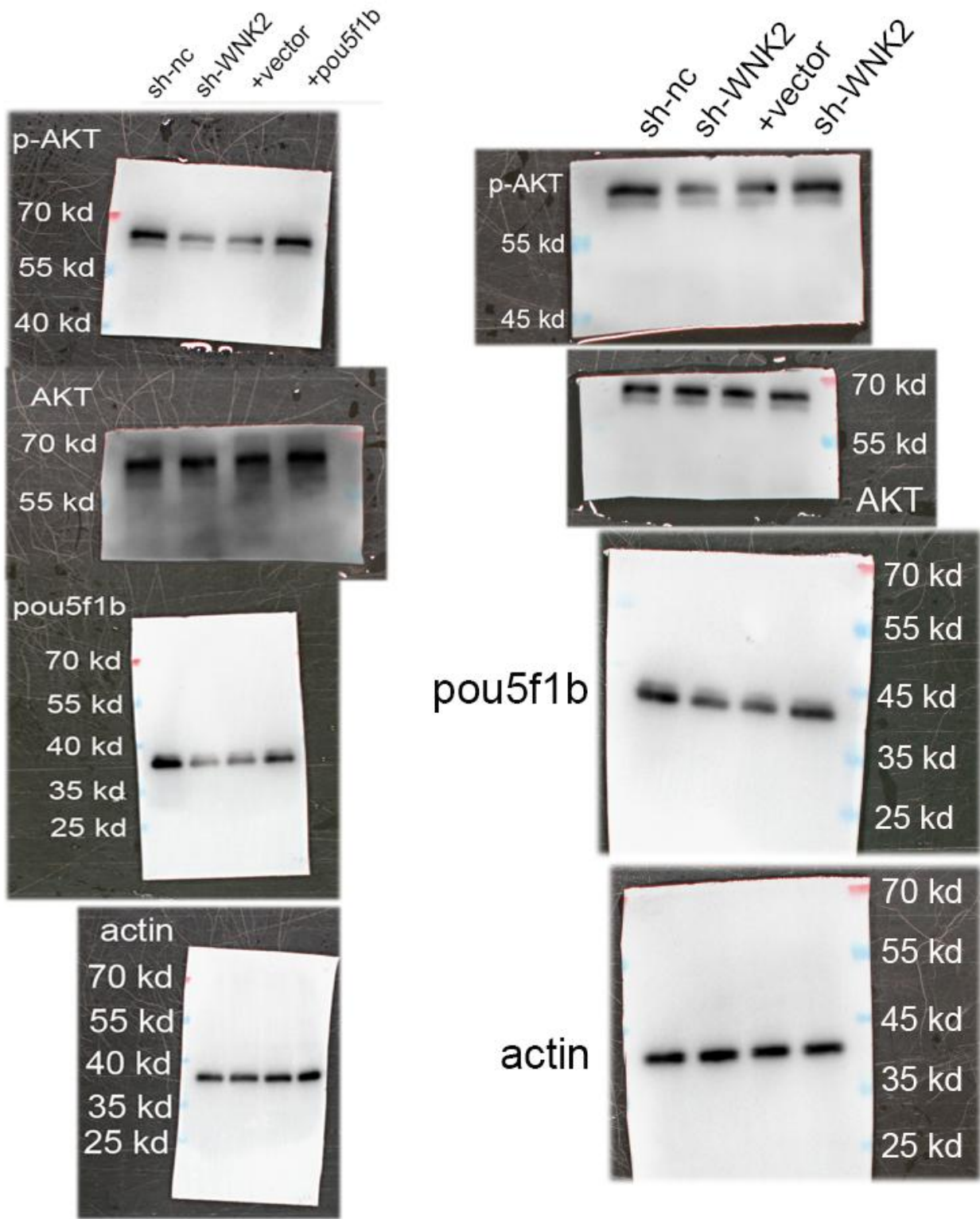

Supplement: S1 File — (PDF) [file pone.0332003.s001.pdf]
