# Supplementary material for: WNK2 may promote ovarian cancer progression by upregulating POU5F1B
Source: PLoS One. 2026 Feb 2;21(2):e0332003. doi: 10.1371/journal.pone.0332003 (PMC12863568; doi:10.1371/journal.pone.0332003)
Supplement: S2 File — (PDF) [file pone.0332003.s002.pdf]

Fig 1b	si_1	si_2	si_3	Con_1	Con_2	Con_3	si	Control
	586.820	597.967	510.151	1272.87	1532.99	1439.86	564.979	1415.24
	257.053	262.907	179.823	522.034	570.691	871.685	233.261	654.803
	0	0.98837	0	3.84555	4.91975	3.06571	0.32945	3.94367
	0	0.98837	0.97730	4.80694	5.90370	6.13143	0.65522	5.61402
	0	0.98837	0.97730	3.84555	3.93580	5.10953	0.65522	4.29696
	2.04823	0.98837	1.95460	8.65250	9.83951	12.2628	1.66373	10.2516
	4.09647	4.94187	2.93190	20.1891	15.7432	25.5476	3.99008	20.4933
	1.02411	0.98837	0.97730	3.84555	3.93580	7.15334	0.99659	4.97823
	0	1.97675	2.93190	6.72972	5.90370	9.19715	1.63621	7.27686
	3.07235	0.98837	0.97730	8.65250	6.88765	5.10953	1.67934	6.88323
	1.02411	2.96512	2.93190	7.69111	12.7913	8.17524	2.30705	9.55257
	2.04823	1.97675	0.97730	7.69111	6.88765	4.08762	1.66743	6.22213
	2.04823	1.97675	0.97730	5.76833	4.91975	6.13143	1.66743	5.60650
	5.12059	2.96512	2.93190	10.5752	10.8234	13.2847	3.67254	11.5611
	12.2894	7.90700	6.84111	27.8802	21.6469	22.4819	9.01251	24.0030
	7.16883	8.89537	6.84111	17.3050	21.6469	21.4600	7.63510	20.1373
	5.12059	3.95350	4.88650	8.65250	12.7913	13.2847	4.65353	11.5762
	6.14471	8.89537	9.77301	17.3050	17.7111	24.5257	8.27103	19.8472
	13.3135	13.8372	13.6822	30.7644	25.5827	39.8543	13.6110	32.0671
	6.14471	5.93025	5.86381	13.4594	14.7592	13.2847	5.97959	13.8344
	3.07235	3.95350	4.88650	10.5752	9.83951	7.15334	3.97078	9.18937
	114.701	137.384	88.9344	258.613	294.201	206.425	113.673	253.080
	8.19295	9.88375	9.77301	23.0733	17.7111	20.4381	9.28324	20.4075
	18.4341	19.7675	20.5233	36.5327	38.3740	51.0953	19.5749	42.0007
	75.7848	67.2095	68.4111	162.474	157.432	126.716	70.4684	148.874
	19.4582	17.7907	15.6368	44.2239	32.4703	33.7228	17.6286	36.8057
	4.09647	2.96512	2.93190	5.76833	7.87160	7.15334	3.33116	6.93109

Fig 1d	Well	Fluor	Target	Content	Sample	Biologic	Cq	Cq Mea
CAOV3	A01	SYBR	large2	Unkn	si-nc		18.4732	18.4732
	A02	SYBR	large2	Unkn	si-nc		19.3910	19.3910
	A03	SYBR	large2	Unkn	si-nc		18.9285	18.9285
	A04	SYBR	GAPDH	Unkn	si-nc		15.9791	15.9791
	A05	SYBR	GAPDH	Unkn	si-nc		15.8642	15.8642
	A06	SYBR	GAPDH	Unkn	si-nc		16.0592	16.0592
	A07	SYBR	TYMP	Unkn	si-nc		27.8209	27.8209
	A08	SYBR	TYMP	Unkn	si-nc		27.7374	27.7374
	A09	SYBR	TYMP	Unkn	si-nc		28.0711	28.0711
	A10	SYBR	GAPDH	Unkn	si-nc		16.1710	16.1710
	A11	SYBR	GAPDH	Unkn	si-nc		15.8372	15.8372

	A12	SYBR	GAPDH	Unkn	si-nc		15.8193	15.8193
	B01	SYBR	large2	Unkn	si-WNK		18.8497	18.8497
	B02	SYBR	large2	Unkn	si-WNK		18.8658	18.8658
	B03	SYBR	large2	Unkn	si-WNK		18.4025	18.4025
	B04	SYBR	GAPDH	Unkn	si-WNK		15.5490	15.5490
	B05	SYBR	GAPDH	Unkn	si-WNK		15.3871	15.3871
	B06	SYBR	GAPDH	Unkn	si-WNK		15.4499	15.4499
	B07	SYBR	TYMP	Unkn	si-WNK		27.6827	27.6827
	B08	SYBR	TYMP	Unkn	si-WNK		27.8717	27.8717
	B09	SYBR	TYMP	Unkn	si-WNK		27.9732	27.9732
	B10	SYBR	GAPDH	Unkn	si-WNK		15.6024	15.6024
	B11	SYBR	GAPDH	Unkn	si-WNK		15.4729	15.4729
	B12	SYBR	GAPDH	Unkn	si-WNK		15.7202	15.7202
	C01	SYBR	CDC42	Unkn	si-nc		18.9565	18.9565
	C02	SYBR	CDC42	Unkn	si-nc		18.5121	18.5121
	C03	SYBR	CDC42	Unkn	si-nc		18.2767	18.2767
	C04	SYBR	pou5f1b	Unkn	si-nc		15.1242	15.1242
	C05	SYBR	pou5f1b	Unkn	si-nc		15.2818	15.2818
	C06	SYBR	pou5f1b	Unkn	si-nc		15.0374	15.0374
	C07	SYBR	trib3	Unkn	si-nc		26.2340	26.2340
	C08	SYBR	trib3	Unkn	si-nc		26.3187	26.3187
	C09	SYBR	trib3	Unkn	si-nc		26.4946	26.4946
	C10	SYBR	GAPDH	Unkn	si-nc		15.2308	15.2308
	C11	SYBR	GAPDH	Unkn	si-nc		15.2009	15.2009
	C12	SYBR	GAPDH	Unkn	si-nc		15.1360	15.1360
	D01	SYBR	CDC42	Unkn	si-WNK		19.2006	19.2006
	D02	SYBR	CDC42	Unkn	si-WNK		19.4038	19.4038
	D03	SYBR	CDC42	Unkn	si-WNK		19.3114	19.3114
	D04	SYBR	pou5f1b	Unkn	si-WNK		16.5866	16.5866
	D05	SYBR	pou5f1b	Unkn	si-WNK		16.5555	16.5555
	D06	SYBR	pou5f1b	Unkn	si-WNK		16.5534	16.5534
	D07	SYBR	trib3	Unkn	si-WNK		27.5934	27.5934
	D08	SYBR	trib3	Unkn	si-WNK		27.4633	27.4633
	D09	SYBR	trib3	Unkn	si-WNK		27.5274	27.5274
	D10	SYBR	GAPDH	Unkn	si-WNK			0
	D11	SYBR	GAPDH	Unkn	si-WNK			0
	D12	SYBR	GAPDH	Unkn	si-WNK			0
Fig 1d	Well	Fluor	Target	Content	Sample	Biologic	Cq	Cq Mea
A2780	A04	SYBR	gapdh	Unkn	vector		21.6979	21.6979
	A05	SYBR	gapdh	Unkn	vector		21.6550	21.6550

	A06	SYBR	gapdh	Unkn	vector		21.4158	21.4158
	A07	SYBR	tymp	Unkn	vector		21.3803	21.3803
	A08	SYBR	tymp	Unkn	vector		21.9412	21.9412
	A09	SYBR	tymp	Unkn	vector		23.0265	23.0265
	A10	SYBR	hes	Unkn	vector		15.1511	15.1511
	A11	SYBR	hes	Unkn	vector		15.0905	15.0905
	A12	SYBR	hes	Unkn	vector		15.3183	15.3183
	B04	SYBR	gapdh	Unkn	wnk2		21.6738	21.6738
	B05	SYBR	gapdh	Unkn	wnk2		21.6098	21.6098
	B06	SYBR	gapdh	Unkn	wnk2		21.5706	21.5706
	B07	SYBR	tymp	Unkn	wnk2		22.0689	22.0689
	B08	SYBR	tymp	Unkn	wnk2		22.1427	22.1427
	B09	SYBR	tymp	Unkn	wnk2		21.5189	21.5189
	B10	SYBR	hes	Unkn	wnk2		14.8580	14.8580
	B11	SYBR	hes	Unkn	wnk2		14.9384	14.9384
	B12	SYBR	hes	Unkn	wnk2		15.1495	15.1495
	C01	SYBR	large2	Unkn	vector		35.7936	35.7936
	C02	SYBR	large2	Unkn	vector		34.3146	34.3146
	C03	SYBR	large2	Unkn	vector		34.4765	34.4765
	C04	SYBR	cdc	Unkn	vector		23.0119	23.0119
	C05	SYBR	cdc	Unkn	vector		23.1196	23.1196
	C06	SYBR	cdc	Unkn	vector		22.8892	22.8892
	C10	SYBR	trib	Unkn	wnk2		14.5287	14.5287
	C11	SYBR	trib	Unkn	wnk2		14.7270	14.7270
	C12	SYBR	trib	Unkn	wnk2		14.8183	14.8183
	D02	SYBR	large2	Unkn	wnk2		34.6680	34.6680
	D03	SYBR	large2	Unkn	wnk2		35.3904	35.3904
	D04	SYBR	cdc	Unkn	wnk2		23.2769	23.2769
	D05	SYBR	cdc	Unkn	wnk2		23.1502	23.1502
	D06	SYBR	cdc	Unkn	wnk2		23.0680	23.0680
	D10	SYBR	trib	Unkn	vector		15.7561	15.7561
	D11	SYBR	trib	Unkn	vector		15.8421	15.8421
	D12	SYBR	trib	Unkn	vector		15.8966	15.8966
	E01	SYBR	pou5f1b	Unkn	vector		38.5441	38.5441
	E02	SYBR	pou5f1b	Unkn	vector		37.3929	37.3929
	E03	SYBR	pou5f1b	Unkn	vector		36.7877	36.7877
	F02	SYBR	pou5f1b	Unkn	wnk2		35.7581	35.7581
	F03	SYBR	pou5f1b	Unkn	wnk2		35.7259	35.7259
Fig 2a	Well	Fluor	Target	Content	Sample	Cq	Cq Mean	
A2780	A01	SYBR	GAPDH	Unkn	si-1	17.5238	17.523879124	

A02	SYBR	GAPDH	Unkn	si-1	17.5163	17.516325453
A03	SYBR	GAPDH	Unkn	si-1	17.4289	17.428977635
A04	SYBR	pou5f1b	Unkn	si-1	33.0958	33.095845103
A05	SYBR	pou5f1b	Unkn	si-1	33.4595	33.459588837
A06	SYBR	pou5f1b	Unkn	si-1	33.3295	33.329569523
B01	SYBR	GAPDH	Unkn	si-2	17.6167	17.616708622
B02	SYBR	GAPDH	Unkn	si-2	17.5916	17.591697862
B03	SYBR	GAPDH	Unkn	si-2	17.6348	17.634860782
B04	SYBR	pou5f1b	Unkn	si-2	32.9878	32.987896665
B05	SYBR	pou5f1b	Unkn	si-2	33.2179	33.217937815
B06	SYBR	pou5f1b	Unkn	si-2	33.2402	33.240238668
C01	SYBR	GAPDH	Unkn	si-3	18.3046	18.304616689
C02	SYBR	GAPDH	Unkn	si-3	18.2744	18.274439357
C03	SYBR	GAPDH	Unkn	si-3	18.1512	18.151215857
C04	SYBR	pou5f1b	Unkn	si-3	33.7726	33.77264621
C05	SYBR	pou5f1b	Unkn	si-3	33.5894	33.589471814
C06	SYBR	pou5f1b	Unkn	si-3	33.5122	33.512235794
D01	SYBR	GAPDH	Unkn	NC	18.2585	18.258506532
D02	SYBR	GAPDH	Unkn	NC	17.9832	17.983247735
D03	SYBR	GAPDH	Unkn	NC	17.9962	17.996297903
D04	SYBR	pou5f1b	Unkn	NC	32.9677	32.967789702
D05	SYBR	pou5f1b	Unkn	NC	32.6676	32.667623511
D06	SYBR	pou5f1b	Unkn	NC	32.5116	32.511697828

Fig 2b	Well	Fluor	Target	Content	Sample	Cq	Cq Mean
caov3	D01	SYBR	WNK2	Unkn	si-3	24.5902	24.590260611
	D02	SYBR	WNK2	Unkn	si-3	24.5860	24.586018234
	D03	SYBR	WNK2	Unkn	si-3	24.4434	24.443411804
	D04	SYBR	GAPDH	Unkn	si-3	15.2877	15.287776847
	D05	SYBR	GAPDH	Unkn	si-3	15.2012	15.201231923
	D06	SYBR	GAPDH	Unkn	si-3	15.2006	15.200639716
	E01	SYBR	WNK2	Unkn	si-1	24.2178	24.217860194
	E02	SYBR	WNK2	Unkn	si-1	24.1326	24.132648112
	E03	SYBR	WNK2	Unkn	si-1	24.0646	24.06464291
	E04	SYBR	GAPDH	Unkn	si-1	14.9008	14.90080832
	E05	SYBR	GAPDH	Unkn	si-1	15.0052	15.005285481
	E06	SYBR	GAPDH	Unkn	si-1	14.9992	14.999266459
	G01	SYBR	WNK2	Unkn	si-2	24.5312	24.53124996
	G02	SYBR	WNK2	Unkn	si-2	24.3727	24.372769473
	G03	SYBR	WNK2	Unkn	si-2	24.3264	24.326488468
	G04	SYBR	GAPDH	Unkn	si-2	14.5302	14.530233358

	G05	SYBR	GAPDH	Unkn	si-2	15.4852	15.485283141
	G06	SYBR	GAPDH	Unkn	si-2	15.5231	15.52312322
	H01	SYBR	WNK2	Unkn	NC	23.8091	24.809148574
	H02	SYBR	WNK2	Unkn	NC	23.8767	23.876735026
	H03	SYBR	WNK2	Unkn	NC	24.0016	24.001608704
	H04	SYBR	GAPDH	Unkn	NC	15.6838	15.683875522
	H05	SYBR	GAPDH	Unkn	NC	15.7238	15.723883484
	H06	SYBR	GAPDH	Unkn	NC	15.8387	15.838701583

Fig 2c	Well	Fluor	Target	Content	Sample	Cq	Cq Mean
a2780	A01	SYBR	POU5F	Unkn	ov	30.4782	30.478295077
	A02	SYBR	POU5F	Unkn	ov	28.5496	28.54962945
	A03	SYBR	POU5F	Unkn	ov	26.7740	26.774066176
	A04	SYBR	gapdh	Unkn	ov	16.4390	16.439065212
	A05	SYBR	gapdh	Unkn	ov	16.4994	16.499443031
	A06	SYBR	gapdh	Unkn	ov	16.3387	16.338742263
	B01	SYBR	POU5F	Unkn	nc	31.6234	31.623458093
	B02	SYBR	POU5F	Unkn	nc	31.5413	31.541386462
	B03	SYBR	POU5F	Unkn	nc	29.2425	29.242567513
	B04	SYBR	gapdh	Unkn	nc	17.4344	17.434405905
	B05	SYBR	gapdh	Unkn	nc	17.2642	17.264230794
	B06	SYBR	gapdh	Unkn	nc	17.2650	17.265029996
Fig 2c	A01	SYBR	POU5F	Unkn	nc	23.8128	23.812804271
caov3	A02	SYBR	POU5F	Unkn	nc	24.0555	24.055507576
	A03	SYBR	POU5F	Unkn	nc	23.8277	23.827739128
	A04	SYBR	gapdh	Unkn	nc	20.8567	20.856725088
	A05	SYBR	gapdh	Unkn	nc	20.8357	20.835773176
	A06	SYBR	gapdh	Unkn	nc	20.7339	20.733974747
	B01	SYBR	POU5F	Unkn	ov	24.3878	24.387859074
	B02	SYBR	POU5F	Unkn	ov	24.1463	24.146370829
	B03	SYBR	POU5F	Unkn	ov	24.3187	24.318793829
	B04	SYBR	gapdh	Unkn	ov	23.0944	23.094428017
	B05	SYBR	gapdh	Unkn	ov	22.9075	22.907517108
	B06	SYBR	gapdh	Unkn	ov	22.9286	22.928694242

Fig 3b	point pc	Cassette ID	tissue ty	Pathological cla	POU5F1B inten
	A01	01A0048-B30-C	cancer	ovarian cancer	98.64
	A02	01A0048-B30-P	oncance	normal tissue	80
	A03	01A0042-B30-C	cancer	ovarian cancer	92.677251
	A04	01A0042-B30-P	oncance	normal tissue	50
	A05	01A0046-B30-C	cancer	ovarian cancer	57

A06	01A0046-B30-P	oncance	normal tissue	4.2074363
A07	01A0014-B30-C	cancer	ovarian cancer	108.14643
A08	01A0014-B30-P	oncance	normal tissue	20
A09	01A0009-B30-C	cancer	ovarian cancer	30
A10	01A0009-B30-P	oncance	normal tissue	16.731942
A11	01A0022-B30-C	cancer	ovarian cancer	60
A12	01A0001-B30-C	cancer	ovarian cancer	73
B01	01A0013-B30-C	cancer	ovarian cancer	39.228895
B02	01A0016-B30-C	cancer	ovarian cancer	89
B03	01A0034-B30-C	cancer	ovarian cancer	98.183023
B04	01A0039-B30-C	cancer	ovarian cancer	29.930374
B05	01A0056-B30-C	cancer	ovarian cancer	107
B06	01A0057-B30-C	cancer	ovarian cancer	93.209158
B07	01A0061-B30-C	cancer	ovarian cancer	80
B08	01A0066-B30-C	cancer	ovarian cancer	60
B09	01A0072-B30-C	cancer	ovarian cancer	92.193517
B10	01A0019-B30-C	cancer	ovarian cancer	41.544661
B11	01A0027-B30-P	cancer	ovarian cancer	40
B12	01A0065-B30-C	cancer	ovarian cancer	88
C01	01A0212-B30-C	cancer	ovarian cancer	67
C02	01A0230-B30-C	cancer	ovarian cancer	98
C03	01A0012-B30-C	cancer	ovarian cancer	40
C04	01A0015-B30-C	cancer	ovarian cancer	85
C05	01A0047-B30-C	cancer	ovarian cancer	80
C06	01A0050-B30-P	cancer	ovarian cancer	123.82494
C07	01A0079-B30-C	cancer	ovarian cancer	100.60638
C08	01A0205-B30-C	cancer	ovarian cancer	85.295149
C09	01A0227-B30-C	cancer	ovarian cancer	92
C10	01A0237-B30-C	cancer	ovarian cancer	60
C11	01A0243-B30-P	cancer	ovarian cancer	40
C12	01A0248-B30-C	cancer	ovarian cancer	102.74757
D01	01A0032-B30-C	cancer	ovarian cancer	65
D02	01A0049-B30-C	cancer	ovarian cancer	103
D03	01A0055-B30-C	cancer	ovarian cancer	95
D04	01A0058-B30-C	cancer	ovarian cancer	104
D05	01A0064-B30-C	cancer	ovarian cancer	108.13883
D06	01A0245-B30-C	cancer	ovarian cancer	79
D07	01A0018-B30-C	cancer	ovarian cancer	40
D08	01A0081-B30-C	cancer	mucinous car	85
D09	01A0085-B30-C	cancer	mucinous car	100
D10	01A0023-B30-C	cancer	mucinous car	40
D11	01A0026-B30-C	cancer	mucinous car	40
D12	01A0041-B30-C	cancer	mucinous car	32

E01	01A0054-B30-C	cancer	mucinous car	40
E02	01A0060-B30-C	cancer	mucinous car	70
E03	01A0074-B30-C	cancer	mucinous car	93
E04	01A0063-B30-C	cancer	mucinous car	110
E05	01A0078-B30-C	cancer	ovarian cancer	101.74519
E06	01A0006-B30-P	cancer	ovarian cancer	80
E07	01A0045-B30-C	cancer	ovarian cancer	94
E08	01A0244-B30-C	cancer	an cancer (clear	58
E09	01A0008-B30-C	cancer	an cancer (clear	52
E10	01A0033-B30-C	cancer	an cancer (clear	72
E11	01A0037-B30-C	cancer	an cancer (clear	50
E12	01A0030-B30-C	cancer	an cancer (clear	40
F01	01A0024-B30-C	cancer	ovarian cancer	40
F02	01A0251-B30-C	cancer	ovarian cancer	120
F03	01A0226-B30-C	cancer	ovarian cancer	108
F04	01A0011-B30-C	cancer	ovarian cancer	59.473211
F05	01A0070-B30-C	cancer	ovarian cancer	80
F06	01A0071-B30-C	cancer	ovarian cancer	85
F07	01A0035-B30-C	cancer	ovarian cancer	82
F08	01A0080-B30-C	cancer	ovarian cancer	100
F09	01A0083-B30-C	cancer	ovarian cancer	109
F10	01A0246-B30-C	cancer	ovarian cancer	60

Fig 3c-d

		number
normal	low exp	3
	modera	2
	high ex|	0
serous	low exp	3
	modera	11
	high ex|	24
mucino	low exp	1
	modera	3
	high ex|	4
other pa	low exp	2
	modera	7
	high ex|	12

Fig 4a

A2780

Well	Fluor	Target	Content	Sample	Cq	Cq Mean
A01	SYBR	GAPDH	Unkn	si-3	20.75	20.75
A02	SYBR	GAPDH	Unkn	si-3	20.8	20.8
A03	SYBR	GAPDH	Unkn	si-3	20.72	20.72
A04	SYBR	POU5F	Unkn	si-3	33.98	33.98

A05	SYBR	POU5F	Unkn	si-3	34.84	34.84
A06	SYBR	POU5F	Unkn	si-3	34.31	34.31
C01	SYBR	GAPDH	Unkn	si-2	21.04	21.04
C02	SYBR	GAPDH	Unkn	si-2	20.85	20.85
C03	SYBR	GAPDH	Unkn	si-2	20.97	20.97
C04	SYBR	POU5F	Unkn	si-2	33.25	33.84
C05	SYBR	POU5F	Unkn	si-2	33.62	33.83
C06	SYBR	POU5F	Unkn	si-2	33.79	34.02
D01	SYBR	GAPDH	Unkn	si-1	22.01	22.59
D02	SYBR	GAPDH	Unkn	si-1	21.73	22.94
D03	SYBR	GAPDH	Unkn	si-1	21.85	22.88
D04	SYBR	POU5F	Unkn	si-1	33.84	33.25
D05	SYBR	POU5F	Unkn	si-1	33.83	33.62
D06	SYBR	POU5F	Unkn	si-1	34.02	33.79
E01	SYBR	GAPDH	Unkn	NC	22.59	22.01
E02	SYBR	GAPDH	Unkn	NC	22.94	21.73
E03	SYBR	GAPDH	Unkn	NC	22.88	21.85
E04	SYBR	POU5F	Unkn	NC	37.4249	37.42
E05	SYBR	POU5F	Unkn	NC	37.4371	37.44
E06	SYBR	POU5F	Unkn	NC	35.7	35.7

CAOV3	Well	Fluor	Target	Content	Sample	Cq	Cq Mean
	A01	SYBR	gapdh	Unkn	si-1	19.94	19.94
	A02	SYBR	gapdh	Unkn	si-1	20	20
	A03	SYBR	gapdh	Unkn	si-1	20.14	20.14
	A04	SYBR	POU5F	Unkn	si-1	31.39	31.39
	A05	SYBR	POU5F	Unkn	si-1	31.99	31.99
	A06	SYBR	POU5F	Unkn	si-1	32.19	32.19
	B01	SYBR	gapdh	Unkn	si-2	21.06	21.06
	B02	SYBR	gapdh	Unkn	si-2	21.56	21.56
	B03	SYBR	gapdh	Unkn	si-2	21.27	21.27
	B04	SYBR	POU5F	Unkn	si-2	31.77	31.77
	B05	SYBR	POU5F	Unkn	si-2	33.64	33.64
	B06	SYBR	POU5F	Unkn	si-2	31.84	31.84
	C01	SYBR	gapdh	Unkn	si-nc	21.44	21.44
	C02	SYBR	gapdh	Unkn	si-nc	21.9	21.9
	C03	SYBR	gapdh	Unkn	si-nc	22.26	22.26
	C04	SYBR	POU5F	Unkn	si-nc	32.61	32.61
	C05	SYBR	POU5F	Unkn	si-nc	31.94	31.94
	C06	SYBR	POU5F	Unkn	si-nc	31.77	31.77
	D01	SYBR	gapdh	Unkn	si-3	16.15	16.15
	D02	SYBR	gapdh	Unkn	si-3	16.55	16.55

	D03	SYBR	gapdh	Unkn	si-3	16.56	16.56	
	D04	SYBR	POU5F	Unkn	si-3	28.22	28.22	
	D05	SYBR	POU5F	Unkn	si-3	28.3	28.3	
	D06	SYBR	POU5F	Unkn	si-3	28.26	28.26	

Fig 4b	a2780		od=450					average
	0h	nc	0.181	0.186	0.186	0.18	0.187	0.184
		si-1	0.186	0.182	0.194	0.182	0.177	0.184
		si-3	0.212	0.192	0.195	0.197	0.19	0.197
	24h	nc	0.192	0.204	0.2	0.216	0.198	0.202
		si-1	0.207	0.212	0.211	0.211	0.2	0.208
		si-3	0.206	0.212	0.198	0.198	0.202	0.203
	48h	nc	0.358	0.341	0.338	0.341	0.383	0.352
		si-1	0.329	0.313	0.31	0.313		0.316
		si-3	0.268	0.276	0.281	0.306	0.278	0.282
	76h	nc	0.85	0.745	0.674	0.686	0.664	0.723
		si-1	0.43	0.434	0.561	0.601	0.465	0.498
		si-3	0.728	0.697	0.722	0.729	0.702	0.716
	4d	nc	1.985	2.143	1.918	2.03		2.019
		si-1	1.081	1.089	1.129	1.502	2.031	1.366
		si-3	1.014	0.972	1.143	0.98	0.959	1.014
	caov3	nc	0.189	0.206	0.21	0.212	0.203	0.204
		si-1	0.2	0.206	0.194	0.203	0.201	0.201
		si-3	0.243	0.241	0.239	0.23	0.241	0.239
		nc	0.287	0.278	0.278	0.3	0.277	0.284
		si-1	0.32	0.253	0.23	0.265		0.267
		si-3	0.314	0.353	0.353	0.331		0.338
		nc	0.865	0.928	0.745	0.727	0.788	0.811
		si-1	0.448	0.45	0.522	0.607	0.483	0.502
		si-3	0.708	0.683	0.669	0.866	0.842	0.754
		nc	1.609	1.446	1.543	1.527	1.535	1.532
		si-1	1.014	0.972	1.143	0.98	0.959	1.014
		si-3	0.672	0.713	0.862	0.899	0.667	0.763
		nc	2.414	2.688	2.697	2.692	2.691	2.636
		si-1	2.284	2.037	1.968	2.059	1.792	2.028
		si-3	2.046	1.729	1.797	1.861	2.168	2.015

Fig 4c	a2780		colony r	Fig 4d	a2780		cell number
		siPOU5	113			siPOU5	326
			105				352
			100				317
		siPOU5	27			siPOU5	168

		23				195
		30				172
	siPOU5	28			siPOU5	147
		51				155
		39				106
caov3	siPOU5	131		caov3	siPOU5	162
		121				172
		115				169
	siPOU5	67			siPOU5	83
		62				90
		59				95
	siPOU5	71			siPOU5	64
		86				88
		81				81

Fig 5a

Well	Fluor	Target	Content	Sample	Cq	Cq Mean
G01	SYBR	POU5F	Unkn	overexp	11.01	11.01
G02	SYBR	POU5F	Unkn	overexp	10.81	10.81
G03	SYBR	POU5F	Unkn	overexp	10.54	10.54
G04	SYBR	GAPDH	Unkn	overexp	20.59	20.59
G05	SYBR	GAPDH	Unkn	overexp	20.56	20.56
G06	SYBR	GAPDH	Unkn	overexp	20.42	20.42
H01	SYBR	POU5F	Unkn	nc	22.03	22.03
H02	SYBR	POU5F	Unkn	nc	21.58	21.58
H03	SYBR	POU5F	Unkn	nc	22.01	22.01
H04	SYBR	GAPDH	Unkn	nc	23.13	23.13
H05	SYBR	GAPDH	Unkn	nc	22.87	22.87
H06	SYBR	GAPDH	Unkn	nc	22.81	22.81

Well	Fluor	Target	Content	Sample	Cq	Cq Mean
G01	SYBR	POU5F	Unkn	overexp	19.19	19.19
G02	SYBR	POU5F	Unkn	overexp	19.13	19.13
G03	SYBR	POU5F	Unkn	overexp	19.2	19.2
G04	SYBR	GAPDH	Unkn	overexp	16.46	16.46
G05	SYBR	GAPDH	Unkn	overexp	16.4	16.4
G06	SYBR	GAPDH	Unkn	overexp	16.31	16.31
H01	SYBR	POU5F	Unkn	nc	26.63	26.63
H02	SYBR	POU5F	Unkn	nc	25.53	25.53
H03	SYBR	POU5F	Unkn	nc	26.13	26.13
H04	SYBR	GAPDH	Unkn	nc	15.95	15.95
H05	SYBR	GAPDH	Unkn	nc	15.92	15.92
H06	SYBR	GAPDH	Unkn	nc	15.81	15.81

Fig 5b	a2780	OD450 value				
	0h	0.249	0.224	0.246	0.244	ov
		0.263	0.258	0.259	0.258	nc
	24h	0.541	0.563	0.563	0.541	ov
		0.587	0.573	0.635	0.635	nc
	48h	0.713	0.741	0.797	0.78	ov
		0.862	0.873	0.877	0.846	nc
	72h	2.238	2.136	2.19	2.059	ov
		1.533	1.67	1.446	1.637	nc
	caov3					
	0h	0.249	0.224	0.246	0.244	nc
		0.263	0.258	0.259	0.258	overexpress
	24h	0.541	0.563	0.563	0.541	nc
		0.587	0.573	0.635	0.635	overexpress
	48h	0.713	0.741	0.797	0.78	nc
		0.862	0.873	0.877	0.846	overexpress
	72h	1.533	1.67	1.446	1.637	nc
		2.381	2.358	2.69	2.359	overexpress

Fig 5c	a2780		colony number
		nc	113
			105
			100
		overexp	27
			23
			30
	caov3	nc	131
			121
			115
		overexp	67
			62
			59
Fig 5d	a2780		cell number
		nc	197
			164
			140
		overexp	190
			248
			212
	caov3	nc	200
			230
			260

overexp	348	
	404	
	331	

Fig 6a

Well	Fluor	Target	Content	Sample	Cq	Cq Mean
C01	SYBR	pou5f1b	Unkn	NC	27.91	27.91
C02	SYBR	pou5f1b	Unkn	NC	27.75	27.75
C03	SYBR	pou5f1b	Unkn	NC	27.53	27.53
C04	SYBR	gapdh	Unkn	NC	17.56	17.56
C05	SYBR	gapdh	Unkn	NC	17.6	17.6
C06	SYBR	gapdh	Unkn	NC	17.3	17.3
C07	SYBR	gapdh	Unkn	NC	17.42	17.42
D01	SYBR	pou5f1b	Unkn	sh-WNK	30.92	30.92
D02	SYBR	pou5f1b	Unkn	sh-WNK	29.14	29.14
D03	SYBR	pou5f1b	Unkn	sh-WNK	30.49	30.49
D04	SYBR	gapdh	Unkn	sh-WNK	18.86	18.86
D05	SYBR	gapdh	Unkn	sh-WNK	18.8	18.8
D06	SYBR	gapdh	Unkn	sh-WNK	18.71	18.71
D07	SYBR	gapdh	Unkn	sh-WNK	18.65	18.65
E01	SYBR	pou5f1b	Unkn	sh-WNK	35.73	35.73
E02	SYBR	pou5f1b	Unkn	sh-WNK	36.05	36.05
E03	SYBR	pou5f1b	Unkn	sh-WNK	35.43	35.43
E04	SYBR	gapdh	Unkn	sh-WNK	24.7	24.7
E05	SYBR	gapdh	Unkn	sh-WNK	24.49	24.49
E06	SYBR	gapdh	Unkn	sh-WNK	24.44	24.44
E07	SYBR	gapdh	Unkn	sh-WNK	24.29	24.29
F01	SYBR	pou5f1b	Unkn	sh-WNK	33.1	33.1
F02	SYBR	pou5f1b	Unkn	sh-WNK	34.09	34.09
F03	SYBR	pou5f1b	Unkn	sh-WNK	33.26	33.26
F04	SYBR	gapdh	Unkn	sh-WNK	24.12	24.12
F05	SYBR	gapdh	Unkn	sh-WNK	24.09	24.09
F06	SYBR	gapdh	Unkn	sh-WNK2+pou5		0
F07	SYBR	gapdh	Unkn	sh-WNK	24.09	24.09

Well	Fluor	Target	Content	Sample	Cq	Cq Mean
A01	SYBR	pou5f1b	Unkn	sh-WNK	23.71	23.71
A02	SYBR	pou5f1b	Unkn	sh-WNK	22.34	22.34
A03	SYBR	pou5f1b	Unkn	sh-WNK	22.47	22.47
A04	SYBR	gapdh	Unkn	sh-WNK	21.32	21.32
A05	SYBR	gapdh	Unkn	sh-WNK	21.38	21.38
A06	SYBR	gapdh	Unkn	sh-WNK	21.27	21.27
B01	SYBR	pou5f1b	Unkn	sh-WNK	23.35	23.35
B02	SYBR	pou5f1b	Unkn	sh-WNK	23.12	23.12

B03	SYBR	pou5f1b	Unkn	sh-WNK	22.34	22.34
B04	SYBR	gapdh	Unkn	sh-WNK	21.25	21.25
B05	SYBR	gapdh	Unkn	sh-WNK	21.24	21.24
B06	SYBR	gapdh	Unkn	sh-WNK	21.19	21.19
C01	SYBR	pou5f1b	Unkn	NC	21.45	21.45
C02	SYBR	pou5f1b	Unkn	NC	20.88	20.88
C03	SYBR	pou5f1b	Unkn	NC	20.41	20.41
C04	SYBR	gapdh	Unkn	NC	21.39	21.39
C05	SYBR	gapdh	Unkn	NC	21.32	21.32
C06	SYBR	gapdh	Unkn	NC	21.36	21.36
D01	SYBR	pou5f1b	Unkn	sh-WNK	20.48	20.48
D02	SYBR	pou5f1b	Unkn	sh-WNK	20.53	20.53
D03	SYBR	pou5f1b	Unkn	sh-WNK	20.66	20.66
D04	SYBR	gapdh	Unkn	sh-WNK	21.83	21.83
D05	SYBR	gapdh	Unkn	sh-WNK	21.84	21.84
D06	SYBR	gapdh	Unkn	sh-WNK	21.65	21.65

Fig 6b		OD450 value						
	a2780	0h	sh-NC	0.201	0.26	0.206	0.259	0.231
			sh-wnk2	0.203	0.21	0.245	0.245	0.226
			sh-wnk2	0.204	0.204	0.201	0.219	0.207
			sh-wnk2	0.204	0.207	0.201	0.202	0.203
		2d	sh-NC	0.507	0.512	0.502	0.52	0.51
			sh-wnk2	0.449	0.434	0.45	0.453	0.446
			sh-wnk2	0.284	0.282	0.352	0.312	0.308
			sh-wnk2	0.449	0.416	0.467	0.438	0.442
		4d	sh-NC	0.686	0.687	0.572	0.563	0.627
			sh-wnk2	0.489	0.518	0.508	0.492	0.502
			sh-wnk2	0.392	0.374	0.359	0.391	0.379
			sh-wnk2	0.464	0.514	0.488	0.451	0.479
		6d	sh-NC	1.193	1.205	1.285	1.199	1.221
			sh-wnk2	0.707	0.715	0.737	0.741	0.725
			sh-wnk2	0.633	0.674	0.705	0.644	0.664
			sh-wnk2	0.926	0.812	0.874	0.892	0.876
		caov3	0h	sh-NC	0.203	0.217	0.209	0.21
				sh-wnk2	0.21	0.214	0.201	0.208
				sh-wnk2	0.214	0.226	0.216	0.219
				sh-wnk2	0.222	0.218	0.224	0.222

	2d	sh-NC	0.559	0.655	0.685	0.633
		sh-wnk:	0.345	0.335	0.349	0.343
		sh-wnk:	0.322	0.32	0.303	0.315
		sh-wnk:	0.455	0.402	0.396	0.418
	4d	sh-NC	0.619	0.718	0.643	0.66
		sh-wnk:	0.482	0.564	0.541	0.529
		sh-wnk:	0.386	0.353	0.428	0.389
		sh-wnk:	0.582	0.521	0.543	0.549
	6d	sh-NC	1.408	1.432	1.214	1.351
		sh-wnk:	1.08	0.976	1.107	1.054
		sh-wnk:	0.605	0.621	0.636	0.62
		sh-wnk:	0.7	0.723	0.722	0.715

Fig 6c

a2780		colony number	
	sh-NC	250	
		221	
		209	
	sh-WNI	120	
		140	
		136	
	sh-WNI	99	
		108	
		91	
	sh-WNI	180	
		206	
		191	
caov3	sh-NC	639	
		584	
		557	
	sh-WNI	302	
		283	
		260	
	sh-WNI	200	
		180	
		163	
	sh-WNI	300	
		312	
		324	

Fig 7

	sh-WNl	sh+pou5f1b
15th da	7.395	12.54
	13.33	13.06
	5.774	15.57
	12.07	6.209
	5.817	4.98
16th da	21.89	47.53
	13.24	92.47
	16.24	63.37
	8.132	22.49
	10.7	59.63
17th da	27.24	115.1
	34.68	99.28
	47.2	14.86
	41.03	216.8
	48.31	59.84
18th da	21.12	46.35
	59.74	180.9
	47.76	217.1
	35.41	136.7
	85.09	125.4

log2Fol	pvalue	gene
-1.3248	3.15E-1	TRIB3
-1.4890	3.8E-1(	SESN2
-3.4663	0.0463(	GRID2IP
-3.0847	0.0331٤	TEX45
-2.6995	0.0784(	PGF
-2.6240	0.0077(	SLC15A1
-2.3607	0.0005	RPL24P2
-2.3184	0.0793	ZNF709
-2.1405	0.0560	LARGE2
-2.0458	0.0629	IL3RA
-2.0440	0.0295	POU5F1B
-1.9051	0.0869	CDK15
-1.7523	0.1203	FLT4
-1.6574	0.0359	INHA
-1.4172	0.0095	CACNA1H
-1.3982	0.0156	PRPH
-1.3140	0.0786	CDC42EP1
-1.2591	0.0301	GRAMD1B
-1.2350	0.0076	TYMP
-1.2105	0.0686	GNMT
-1.2097	0.1398	ATF4P3
-1.1553	1.18E-(	HES6
-1.1357	0.0393	NXPH3
-1.0996	0.0059	TMEM91
-1.0801	6.04E-(	SLC6A9
-1.0638	0.0115	ICAM5
-1.0584	0.2443	LDB3

ın
204599
)70538
571385
I23201
252411
239869
)74438
120829
I18776
)96989
247039

75258
731409
00427
99505
20937
31528
217
66032
52811
03306
26127
13848
69048
56332
20921
44254
98752
29118
67107
93272
44317
04141
42751
0468
94797
53776
02092
61736
93994
99538
0728
75458
20528
06953

n
970508
55684

387253
364119
294048
518014
146185
572976
397168
348649
337376
551208
981688
706339
908176
015714
407611
524028
571422
529397
521696
944663
541019
292199
791711
049986
318892
090265
438629
907566
224126
015534
156576
18562
63131
165716
960046
743632
192826
993418

sity by imageJ

87

99
78
22
49
15
24
93
08
31
08
65
95
33
53

49
29
