# Supplementary material for: WNK2 may promote ovarian cancer progression by upregulating POU5F1B
Source: PLoS One. 2026 Feb 2;21(2):e0332003. doi: 10.1371/journal.pone.0332003 (PMC12863568; doi:10.1371/journal.pone.0332003)
Supplement: Attachment — Submitted filename: Responses to reviewers.pdf [file pone.0332003.s003.pdf]

We extend our deepest appreciation to the reviewers and the editors for their time and insightful comments, which are immensely helpful for improving our manuscript.

Response to editor.

1. We have adopted your suggestion to deposit our laboratory protocols in protocols.io. (DOI dx.doi.org/10.17504/protocols.io.q26g7nn81lwz/v1)
2. We have formatted the article according to the guidelines.
3. We have stated the role of the financial disclosure: "Key Project of Chongqing Technology Innovation and Application Development Special Project (CSTB2022TIAD-KPX0154)" in the study, and included this amended role of Funder statement in the cover letter.
4. The raw data and original uncropped and unadjusted images have been uploaded to the supplementary materials.
5. We have deleted the ethics statement from any other section besides Materials and Methods.
6. In cover letter, we note the blot/gel image data are provided in Supporting Information.
7. We have uploaded the figure files to the Preflight Analysis and Conversion Engine (PACE) digital diagnostic tool, to ensure that figures meet PLOS requirements.

Response to Reviewer 1

1. The manuscript has underwent professional English language editing by Genesis Technology Communication, Co. Ltd.
2. We have properly formatted Table 1 for easier clarity and readability.
3. We have corrected the text in subsection 3.1 and the legend of Fig.1. We also defined the acronyms (e.g., GBM and others) in the manuscript.
4. We have added the description of Figures 3c–d in the main text.
5. We have improved the layout of the graphics for better interpretation.

Response to Reviewer 2

1. We have added the clinical implications or xenograft sample size in the abstract.
2. We have carefully structured the introduction to be more logical.
3. We appreciate your suggestion very much. The AKT kinase is a cornerstone of oncogenic signaling, whose activation drives cancer progression through multiple mechanisms. The finding that POU5F1B can attenuate WNK2-induced AKT activation underscores its pivotal role as a central modulator of this pathway. We found that WNK2 promotes the transcription of POU5F1B, suggesting that it likely activates a specific transcription factor responsible for POU5F1B expression. Because WNK2 is a protein kinase, not a transcription factor. We design phosphorylation sequencing on WNK2, followed by co-immunoprecipitation (Co-IP) of POU5F1B and mass spectrometry. The ultimate goal is to identify and validate the interacting proteins between them. This set of experiments is being planned as the foundation for

our next-step work. The work plan for this part is outlined in the Discussion section. By the way, RAS is a fundamental family of small GTPase proteins that act as crucial molecular switches, Given that RAS is not a transcription factor too, and its regulation of POU5F1B is indirect, we have decided to remove this set of experiments from the manuscript to maintain a focused narrative on direct regulatory mechanisms.

4. We appreciate your suggestion, to demonstrate that POU5F1B impacts the activation of AKT by WNK2, we assessed AKT activity following WNK2 knockdown and POU5F1B overexpression. Cells were transfected with plasmids for 48 hours to achieve successful gene overexpression. This is consistent with conditions used in published studies investigating gene's function. While a systematic dose- and time-response analysis is undoubtedly valuable, However, we found it challenging to achieve the ideal dose-/time-dependent effects under our experimental conditions.

5. We have incorporated discussions on the potential of WNK2 and POU5F1B as biomarkers and therapeutic targets in our manuscript.
